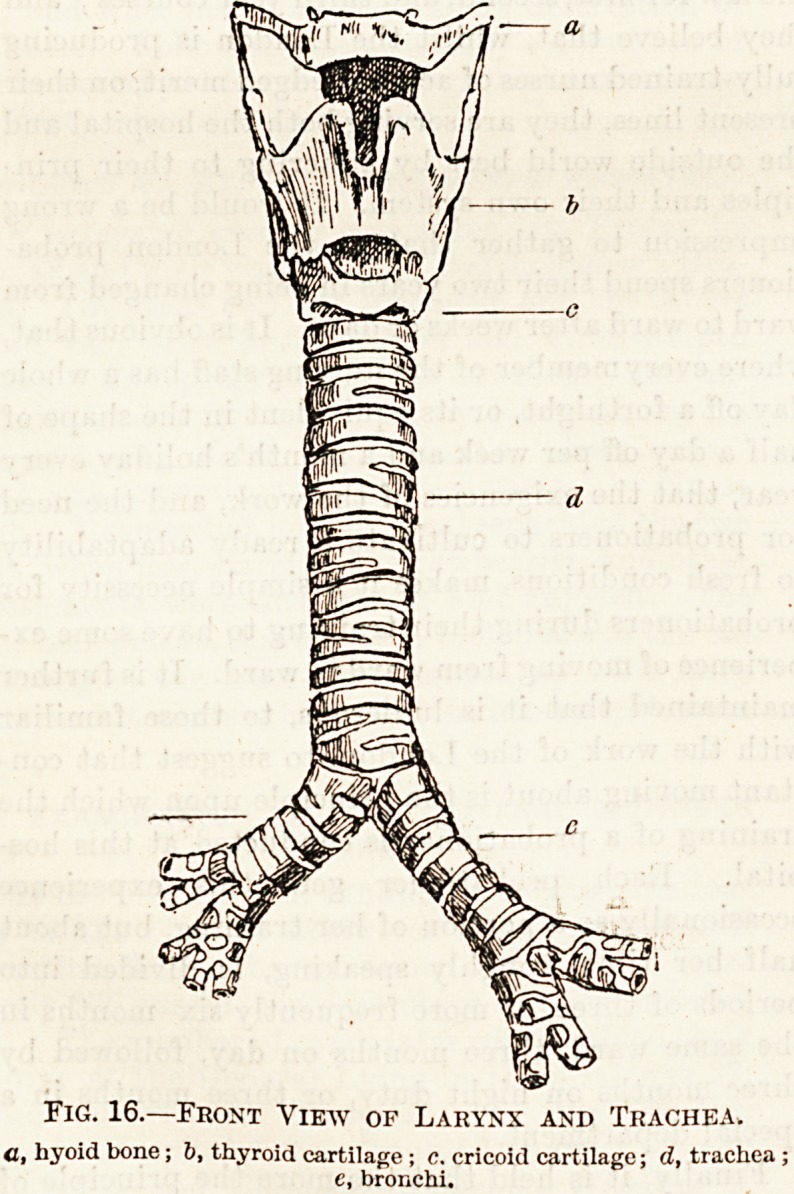# "The Hospital" Nursing Section

**Published:** 1906-07-07

**Authors:** 


### The Hospital.

### Nursing Section.

Contributions for "THE HOSPITAL," should be addressed to the EDITOR, "THE HOSPITAL" NURSING SECTION, 28 & 29 Southampton Street, Strand, London, W.C.

No. 1,033.—VOL. XL. SATURDAY, JULY 7 1906.

### Notes on News from the Nursing World.

### THE KING AND THE AGED NURSE.

THERE is one inhabitant of Derby, at any rate, who will not forget the visit of the King to the town last week. During his inspection of the Army veterans, his Majesty noticed that with them on parade was an old lady. Inquiries were made, and it was ascertained that she was Mrs. Milne, the widow of a staff-sergeant, who served in the Crimea. She herself was a nurse first at Varna, and afterwards at Scutari, before the arrival of Miss Florence Nightingale. These facts being communicated to his Majesty, he desired that Mrs. Milne should be presented to him. The King having shaken hands with her, and asked after her health, she begged his acceptance of a rose she was wearing. With the words "Thank you very much" the Sovereign took the flower and kept it in his possession during his subsequent tour through the sheds of the Royal Agricultural Society's show, thus affording the donor the utmost pleasure. Anxious as she was to attend the ceremony, the veteran nurse never expected to have the honour of being presented to the King, and she deeply felt his kindness.

### THE SECRETARY FOR WAR AND THE ARMY NURSING SERVICE.

ON Monday Mr. Morton, the member for Sutherland, asked the Secretary of State for War whether he proposes to make reductions in Queen Alexandra's Imperial Military Nursing Service, "seeing that the numbers employed in military hospitals are far in excess of the requirements of the sick, and that much money could be thereby saved." Mr. Haldane, in reply, said that the establishment of the service was fixed to provide for the efficient nursing of the sick in military hospitals at home and abroad, and to establish in connection with these hospitals nurse-training schools for the men of the Royal Army Medical Corps. Increased responsibilities in both respects have, he mentioned, been delegated to the nursing service, and having stated that the distribution of the staffs has been decided upon by the civil matrons and other members of the Nursing Board, in whose hands all authority relating to Army nursing is vested, he assured the House that the numbers are not, in the opinion of his expert advisers, in excess of the requirements. He added, "It must be remembered that the standards of the medical care and treatment which are requisite for the sick have risen during the last few years, and that the improvement is resulting not only in more humane methods, but in a saving of public money, which is not less substantial because it is indirect." Here then is conclusive confirmation from the lips of the Secretary of State for War of the opinions expressed in these columns a fortnight ago, and a crushing answer to the enemies of progress. Meanwhile, the *Broad Arrow* continues to allow its columns to be used by the advocates of retrogression in the system of Army nursing, though no further editorial comments have been made. The annonymous correspondents are, however, so clearly of the same mind as the author of the original attack that their letters might easily have been written by the same person. We notice that one of these correspondents who veils his identity under a famous Latin phrase, is very indignant with us because we presumed to say that the only cause of dissatisfaction on the part of the Army surgeons is that the total of female nurses is not sufficient. Nevertheless, we repeat the statement, with the addition that Army surgeons, exclusive of those who want to put the clock back, would be glad if the proportion of educated female nurses to patients in military hospitals could be greater than it is. Our observation that it is open to discontented nurses to resign is termed by this correspondent "nonsense," because as he affirms, "it is no secret that resignations are discouraged." We have yet to hear of any hospitals where resignations are encouraged, except in the case of incapables, and even "*Magna est veritas, et prævalebit*" does not insinuate that the nurses attached to Queen Alexandra's Military Service are incapable of doing their work. His grievance is that they are preferred to males.

### NURSING THE INJURED AT SALISBURY.

NEWS was brought to the Infirmary at Salisbury by an official of the railway of the terrible extent of the accident which occurred on Sunday. The house surgeons at once made preparations to receive the injured. The night sister gave directions to each night nurse to prepare all available beds and to be ready in case of any emergency which might arise. The alarm was next given to the ward sisters and senior nurses, who within ten minutes were at their posts. All the medical men in the town were working at the scene of the disaster, and as the victims were extricated they were sent on stretchers to the Infirmary, where they were received by the house surgeons and distributed to the different wards. In many cases the injuries were so terribly severe that little could be done, but the courage and patience shown by all was the subject of much comment. The prompt and admirable manner in which the entire staff responded to the call upon them, and the care and attention manifested by the nurses, has already been warmly recognised by the patients and their friends on both sides of the Atlantic.

### ST. GEORGE'S HOSPITAL AND PRIVATE NURSING.

Although it has been decided to start a staff of private nurses in connection with St. George's Hospital, London, no definite arrangements have yet been made, except that the Home itself has been selected. It adjoins the Nurses' Home, and the lease only came into the possession of the hospital last month. The decision of the Governors has given satisfaction to the nursing staff, as many at the end of their training will now be able, if they wish to take up private nursing, to attach themselves to their old school. As to the question of remuneration—which elsewhere has been almost condemned before it is known—it is evidently anticipated that the conditions will be on a just and liberal scale, because, as a member of the staff said, "Those of us who cannot afford to remain hospital nurses will in future not have to go outside for private work."

### THE NEW MATRON OF ST. MARY'S HOSPITAL, PADDINGTON.

The Governors of St. Mary's Hospital, Paddington, have chosen out of a number of applicants, as the new matron, Miss Mary E. Davies, at present matron of Queen Charlotte's Lying-in Hospital. The appointment is somewhat of a surprise, but Miss Davies has a highly creditable record. Trained at King's College and the City of London Lying-in Hospital, she was appointed, on the completion of her training, ward sister at University College Hospital, subsequently becoming assistant matron and ward sister. She has since been matron of Queen Charlotte's Lying-in Hospital. It was under the auspices of Miss McCord that the handsome Nurses' Home at Queen Charlotte's Hospital was opened in 1899 by the present Princess of Wales, but the reputation of the training school has been maintained since Miss Davies took the reins.

### THE NEW MATERNITY DEPARTMENT AT THE LONDON HOSPITAL.

Although no official statement appears to have been made, we are able to state that the new maternity department at the London Hospital has been recognised by the Central Midwives Board as a training school for midwives.

### NIGHT NURSES WITHOUT FOOD.

An extraordinary statement was made by one of the Aston Guardians at their last meeting, and it was to the effect that the night nurses "often have to go without food during the whole night owing to the pressure of work." This is an intolerable condition of things and we are surprised that, as we know it to be true, the proposal of the Infirmary Committee to appoint six additional probationers was rejected in favour of an amendment reducing the increase to three. It is obvious that if the night nurses at Aston Infirmary do not get their food at regular times, and plenty of it, they cannot be in a fit condition to discharge their duties in the wards properly. We regard it as a matter of vital importance to both patients and nurses that the staff of the latter in an institution should always be large enough to allow of adequate time for meals both for day and for night nurses.

### THREE YEARS' TRAINING AT THE MILLER HOSPITAL.

The General Committee of the Miller Hospital and Royal Kent Dispensary, Greenwich, at a meeting recently held, adopted a course of three years' training with salary during the whole time and uniform instead of the present training of only two years, with no salary or uniform the first year. It has thus been recognised by the authorities that only a nurse with three years' training has any chance of obtaining the higher appointments or of being accepted by the various Boards as a fully-qualified nurse. Instruction is to be given and examinations are to be held in nursing, practical and theoretical, elementary physiology and anatomy, the theory and practical uses of electricity and *x*-rays, and pharmaceutics appertaining to nursing. We congratulate Miss Watts, the matron, who only took up her duties in February last, upon this new and welcome departure.

### GUARDIANS AND DISCIPLINE.

Last week we briefly announced that the nursing staff at Kingston Poor-law Infirmary had been increased by the addition of three staff nurses and four probationers. This addition was the result of a consultation between the Infirmary Committee and the medical officer and matron; and it ensures two nurses being always on duty on each floor at one time. The question of the compulsory resignation of the two nurses, whose statement of their case has already appeared in our columns, was also referred to at the meeting of guardians, and it was stated by the chairman that sufficient time was allowed the nurses for tea after dinner, and that there was every accommodation for making it. There was, therefore, no excuse for the breach of discipline, on which we have commented, and we think that the guardians wisely declined to listen to a proposal to relax the rules because they were lately transgressed.

### A REFLECTION ON THE COURT SUBURB.

Princess Louise, Duchess of Argyll, presided at the annual meeting of the Kensington District Nursing Association, which was held on Wednesday afternoon last week in the Kensington Vicarage gardens. The adoption of the report was moved by Dr. Samuel West, and seconded by Archdeacon Sinclair. In Kensington during the past year 1,246 cases were attended by eight nurses and the superintendent, but in spite of excellent work we regret to learn that there was a deficit of over £100. This is not at all creditable to the Court suburb, in which the rich are so strongly represented. They ought, at least, to take care that enough money is forthcoming to pay for the nursing of the sick at their own doors.

### THE PENSION FUND AND COVENTRY NURSES.

A meeting of nurses was held on Wednesday, June 27th, at the Coventry and Warwickshire Hospital, Coventry, to hear an address from Mr. Louis Dick on the benefits and rules of the Royal National Pension Fund. Mr. Alfred Herbert, chairman, presided, and there were also present Mr. E. E. Crisp (Secretary), Miss Hoadley (matron), Miss Davidson (matron, City Hospital), Miss Wing (lady superintendent, Nursing Institution), Mrs. Simmons (of the Medical and Surgical Home), also nurses from various institutions in the city. The chairman having strongly commended the objects of the Pension Fund, Mr. Dick delivered an instructive and interesting address. A hearty vote of thanks was subsequently proposed to him by the chairman.

### THE CANON'S FEAR OF A NURSE.

At a meeting held in Holborn Town Hall under the auspices of the Guild of St. Barnabas, Canon Newbolt confessed that he spoke with trepidation. He admitted that he was " a little afraid of addressing nurses," because " there was a possibility that when he stood up before them he might be looked upon as a case." He asked his hearers to forget that he was a possible case, and to look upon him as a human being. The numerous nurses present were much amused both with this appeal of Canon Newbolt, and with his subsequent short description of the nurse. Following the schoolboy's definition of salt, he portrayed her as " a thing which makes sickness nasty when she is not there."

### CONCERT AT ST. BARTHOLOMEW'S HOSPITAL.

An enjoyable evening was spent last Friday by the staff and their visitors at St. Bartholomew's Hospital. The great hall was thronged, and the nurses looked very trim in their blue and white-striped dresses. The programme was excellent, and included " Sealed Orders," sung by Nurse Henry; a four-part song of Sir V. Stanford's, " Diaphenia," which was encored; and " La Seneriata," by Nurse Pugh. A somewhat long interval separated the two parts, when ices, claret cup, strawberries, and other refreshments were distributed and enjoyed. The night being clear, this function took place in the old quadrangle; the trees were lighted up with fairy lights, and it was late before all the good-nights were said.

### THE EXAMINATION OF THE CENTRAL MIDWIVES BOARD.

The list of successful candidates at the examination of the Central Midwives Board last month shows that the number examined was 376. Of these exactly 300 passed, the percentage of failures being 20.2. The number of candidates who received their training at the General Lying-in Hospital was 22, Queen Charlotte's Hospital 14, the British Lying-in Hospital 7, the City of London Lying-in Hospital 5, and Clapham Maternity Hospital 3. But the Maternity Charity Plaistow, stands first on the list with 26. Among other institutions four were trained at Guy's Institution, three at the London Hospital, six at the Royal Infirmary, Bristol, 15 at St. Mary's Hospital, Manchester, five at the Edinburgh Maternity Hospital, and three at the Rotunda Hospital, Dublin. Of the Poor-law institutions, seven were trained at Belfast Union Maternity Hospital, three at the West Ham Union Infirmary, five at the Liverpool Workhouse, Liverpool, two at Kensington Union Infirmary, and one at Croydon Union Infirmary. The single candidate who was trained at Manchester Chorlton Union failed, and the percentage of failures was 75 per cent. in the case of candidates trained by the Cardiff branch of Queen Victoria's Jubilee Nurses' Institute, and 60 per cent. in that of those trained by the Kingswood Cottage Nurses' Home. The comparatively small number of failures points to the conclusion that the general tone of the teaching is excellent, or that the examination is not unduly severe.

### £10,000 FOR A NURSING INSTITUTION.

The munificent sum of £10,000 has been left, under the will of Mr. F. W. Webb, for many years superintendent engineer of the London and North-Western Railway, who died last month, to found a nursing institution in Crewe, or the immediate vicinity. Part of the sum is to be applied in the purchase of a site and the erection of a building or the adaptation of an existing building, and the balance is to form a fund for the maintenance and upkeep of the institution, so that one or more nurses shall at all times be available for the purpose of gratuitous attendance on persons of the poorer classes in Crewe.

### ST. LUKE'S HOME FOR THE DYING POOR.

At a recent drawing-room meeting given by permission of Mr. and Mrs. R. W. Perks at their residence in Kensington Palace Gardens in aid of St. Luke's Home for the Dying Poor at Bayswater, Mr. Howard Barrett, M.R.C.S., founder of the Home, referred to the great need of separate quarters for the nursing staff. This was emphasised by Mr. Pearce-Gould, F.R.C.S., who also commented on the value of such homes as segregation camps, and said that it was most undesirable for the nurses to sleep in the same house as patients suffering from phthisis in acute and advanced stages or cancer in its last stage, Mr. Arnold White said that the nurses engaged in this work had touched the ideal of the nursing profession. Sister Lily, of the West London Mission, dwelt pathetically on the sorrowful cases and traced their regeneration of heart and conduct back to the tender ministrations of those who nursed them during their often protracted last illness.

### SHORT ITEMS.

Miss M. J. Harrison has been appointed a nursing sister in Queen Alexandra's Imperial Military Nursing Service for India.—The Countess of Craven will lay the foundation-stone of the new Nurses' Home of the Coventry and Warwickshire Hospital on July 26th. The Home is to contain 36 bedrooms, sitting-rooms for the sisters and nurses, and reading-rooms, Home sisters' quarters, and a room for the nurses to see their friends.—A feature of the " Summer School " of the Women's Total Abstinence Union, held at Weston-super-Mare last month, was an address by Dr. Annie McCall on " The Physiological Reasons for Total Abstinence as a Habit," to which nurses were specially invited.

### The Nursing Outlook.

"From magnanimity, all fears above;
From nobler recompense, above applause,
Which owes to man's short outlook all its charm."

### METHODS OF TRAINING.

WE emphasised last week that the purpose of the three articles we have published on a great nurse-training school was not criticism, but helpfulness through the eyes of experience. We hope, with the co-operation of the authorities, to bring out the varying methods of training pursued by the greater English schools, and we have pleasure, as a commencement, in giving the views of the system pursued at the London from the point of view of one of its greatest upholders. The London Hospital has no desire to lay down the law for other hospitals, if they find a longer period of training necessary. But the London Hospital affords exceptional opportunities for varied and abundant experience. Probationers get a careful preliminary training at Tredegar House, where they are taught the elementary duties of nursing throughly and individually, in addition to sick cookery, bandaging and other matters.

When they enter the wards they are equipped for their work, and prepared to be taught how to apply the practical knowledge of elementary nursing duties which has already been imparted to them. During their two years they attend continuous courses of lectures and classes for ten months consecutively, and then pass a thorough examination. This theoretical instruction, combined with their practical training, and the varied experience of the medical and surgical nursing of men, women, and children, which is carefully provided for each probationer, makes all of those, who bring the right personal qualifications to the work, technically efficient by the expiration of their two years. They gain increased experience, under continued supervision, during the whole of the four years which have to be completed before their certificate is fully filled up. It appears that at the London no one wishes to underrate the value of experience. A recently qualified man is, no doubt, worth more after he has filled his term as house surgeon and house physician, and a London nurse, in the same way, when she gets her certificate of training, is in the right position to undertake increased responsibilities.

Again, from every point of view it is held to be short-sighted to keep a capable woman back from undertaking duties which she is perfectly qualified to fill, because, perhaps in smaller hospitals, where the work is not organised in such a way as to secure careful opportunities for training, a similar amount of technical knowledge and experience could not be obtained in the same period. When a child knows its alphabet correctly it is of no use to try to keep its attention fixed upon the letters; it wants to go forward, and this is emphatically the case with intelligent women, who wish to become skilled nurses. It is very demoralising in work to be merely putting in time.

The view just advanced is held to prove that the London authorities act on carefully considered principles, of which the common sense appears to be fully justified by the results. The London Hospital authorities do not go about urging that anybody can become a highly trained nurse in any hospital, however limited its opportunities, in any number of years, long or short, but the opportunities and the means taken to enable probationers to profit by them have to be estimated as a whole, before any reasonable conclusion can be arrived at. They deem it a mistake to try to make nursing education analogous with medical education, and to lay down the law for first, second, and third year courses; and they believe that, whilst the London is producing fully-trained nurses of acknowledged merit, on their present lines, they are serving both the hospital and the outside world best by adhering to their principles and their own system. It would be a wrong impression to gather that at the London probationers spend their two years in being changed from ward to ward after weeks or days. It is obvious that, where every member of the nursing staff has a whole day off a fortnight, or its equivalent in the shape of half a day off per week and a month's holiday every year, that the exigencies of the work, and the need for probationers to cultivate a ready adaptability to fresh conditions, makes it a simple necessity for probationers during their training to have some experience of moving from ward to ward. It is further maintained that it is ludicrous, to those familiar with the work of the London, to suggest that constant moving about is the principle upon which the training of a probationer is conducted at this hospital. Each probationer gets this experience occasionally as a portion of her training, but about half her time, roughly speaking, is divided into periods of three, or more frequently six months in the same ward, three months on day, followed by three months on night duty, or three months in a special department.

Finally, it is held that the more the principle of allowing all training schools to retain their freedom to develop on their own lines is upheld, the greater will be the service to nurses and nursing. In this connection those interested may find it helpful to re-peruse some of the articles published in the "Outlook" last year from September 23rd onwards.

### The Care and Nursing of the Insane.

By PERCY J. BAILY, M.B., C.M.Edin., Medical Superintendent of Hanwell Asylum.

### I.—ANATOMY AND PHYSIOLOGY.

(*Continued from page* 175.)

We may now turn our attention to the function of respiration and the anatomical and mechanical arrangements by which it is brought about. In doing so we will arrange the matter under the following headings:—

1. *The organs of respirations* which include the *air passages* and the *lungs*.
2. The formation of the *air-tight box* or *chest cavity* (thorax), in which the lungs are contained.
3. The *act of respiration* and its stages.
4. The *mechanism* of *respiration*.
5. We must renew our acquaintance with the respiratory function of the blood.
6. We must note the differences between the *air we inspire* and *that which* we *expire*.

I. *The Organs of Respiration.*—The air passages are (1) *The Nose.* We normally breathe through the nose, and not through the mouth. As the air passes through the nose it is led over a considerable

FIG. 16.—FRONT VIEW OF LARYNX AND TRACHEA.
*a*, hyoid bone; *b*, thyroid cartilage; *c*, cricoid cartilage; *d*, trachea; *e*, bronchi.

surface of mucous membrane which lines the interior of this as well as the other portions of the air passages. It is thus warmed, and much of the dust which is always present in the air adheres to the mucous secretion in the nose, and is thus prevented from entering the lungs. The nose communicates behind with (2) the pharynx by two openings called the posterior nares, which open into the pharynx above the soft palate. (3) The Larynx (Fig. 16).—This is that portion of the air passages which contains the vocal cords. Its walls are made chiefly of pieces of cartilage, more or less movable on each other and connected together by membranes and muscles. It forms the prominence in front of the neck immediately below the chin, commonly called "Adam's Apple." The mucous membrane lining the larynx is arranged so as to form a sharp, straight, and slightly stretched fold on either side which runs from before backwards. These sharp edges are the vocal cords. They lie parallel with one another, leaving a narrow chink between them through which the air passes. This narrow chink is called the *glottis.* It is closed during the act of swallowing in the manner we have already seen. The epiglottis projects upwards behind the tongue immediately in front of the glottis. During the swallowing of fluids it may help to divert the stream to each side of the opening of the glottis. (4) *The trachea or windpipe* passes through the neck into the thorax. Its walls are composed of incomplete hoops of cartilage, which are connected together by an elastic membrane. A piece of the hoop is wanting at the back, so that its posterior wall is composed only of membrane. The trachea lies in front of and against the œsophagus. After entering the thorax the trachea divides into two branches. (5) The *Right and Left Bronchus*, each of which passes into the lung of its own side. Within the lung each bronchus divides up into a great number of branches, which become smaller and smaller as they pass deeper and deeper into the substance of the lung. These are called the *Bronchioles* or *Bronchial tubes*. Before terminating in the air cells, as we shall presently see, they become so minute that they cannot be seen without the aid of a microscope. The walls of the bronchi and of their larger branches contain cartilaginous hoops like those of the trachea, but in the smaller air tubes (bronchioles) these disappear, and their walls are entirely membranous.

*The Lungs* are two in number. They together completely fill the cavity of the chest (except such parts as are occupied by the heart, the great blood-vessels, the trachea, and the two main bronchi and the œsophagus), and form, as it were, a perfect mould of the interior of this cavity. They are elastic, spongy organs, and, being filled with air, they are very light and float when placed in water. Each lung is more or less conical in shape, the smaller end or *apex* fitting into the narrow upper end of the thorax behind the clavicle and beneath the upper ribs. The broad end or *base* of the lung occupies the lower part of the chest, and rests upon the upper convex surface of the diaphragm. The outer surface of the lungs is everywhere in contact with the chest wall, their inner surfaces look towards each other and over-lap the heart, which lies between them. It is on the inner surface that the bronchus of each side enters the lung, and here also enter and leave the great blood-vessels which pass between the lungs and the heart—these are, as we already know, a branch of the pulmonary artery which conveys the venous blood from the right ventricle to the lungs, and two pulmonary veins which convey the oxygenated arterial blood from the lungs to the left auricle. This part of the lung where the bronchus and pulmonary artery enter, and the pulmonary veins leave it, is called the *Root* of the lung.

The surface of the lungs is everywhere covered by a closely adherent, thin, shining membrane called the *Pleura.* At the root of the lung on each side it is reflected on to the inner surface of the walls of the thorax, which it also completely lines. Thus each lung lies within a sort of bag, just as we have seen is the case with the heart, with this difference, however, that whereas the outer layer of the pericardium is a loose bag, the outer layer of the pleura is closely and firmly attached to the inner surface of the thoracic wall and the upper surface of the diaphragm.

Each lung consists of an enormous number of little elongated dilatations, whose walls are composed of an elastic membrane. These dilatations are the terminations of the minute bronchial tubes. They are like little elastic bladders, but their walls, instead of being smooth and even, are made up of numerous little indentations or sacs—*air cells,* as they are called—whose mouths open irregularly into the cavity of the dilatation. If it were possible to isolate one of these dilatations and blow it up with air it would look something like a bunch of grapes, only the different sacs would not be so distinct from one another as the individual grapes. The walls of the air cells are very thin, and are composed of a delicate and highly elastic membrane *which carries the capillary blood-vessels* into which the pulmonary artery pours its blood. The object of this arrangement is to bring the impure venous blood of the pulmonay artery into as close contact with the air as possible in order that it may gain oxygen from the air and get rid of the carbonic acid gas which it contains.

During its passage through the pulmonary capillaries the blood is separated from the air in the air cells only by the thin walls of the capillaries themselves and the epithelial lining of the air cells. These form a delicate membrane through which both oxygen and carbonic acid are, in common with all gases, enabled to pass by what is called the property of diffusion.

We see then that the air in the air cells of the lungs gives up its oxygen to the blood contained in the pulmonary capillaries, and receives from this same blood carbonic acid in exchange. It is obvious, therefore, that the air in the air cells would soon become so charged with carbonic acid and so deficient in oxygen as to be useless for the purposes of respiration if there were not some provision made to ensure its being frequently changed. This changing of the air in the air cells is brought about by the *movements* of *respiration.*

### The Nurses' Clinic.

### NOSE AND THROAT CASES—BEFORE OPERATION.

Nose and throat operations are sometimes performed without anæsthetics, though some local or general anæsthetic is generally administered to deaden the pain for the patient, and to render the operation easier for the surgeon.

Local anæsthesia may be produced by drugs which temporarily deaden pain, or for such small operations as tonsillotomy, uvulotomy, and for opening an abscess quinsy. Heat or cold may produce sufficient anæsthesia for these cases.

Heat can be applied by special hot-air apparatus, or by hot-water douche (temperature 110°, 115° to 120° F.). A hot application, to be of any good, must first cause a sharp burning sensation, which passes off soon and leaves the membrane partially deadened to sensation.

The temperature of cold douches varies from 38° to 40° F. for the nose; or ice may be applied to the nose, palate, or neck, as the case may require. Cold is a more effectual anæsthetic than heat, and the effect lasts longer.

Cocaine, eucaine, carbolic acid (10 per cent.) are generally used for local anæsthesia. Cocaine cannot be taken well by some patients, bad effects coming on very rapidly, and sometimes being most alarming. The bad effects arise mostly after cocaine has been hypodermically injected, though it may also occur by absorption into the general system when applied to the mouth or throat. Signs of cocaine poisoning are pallid face, dull eyes, extreme exhaustion, muscular relaxation, pulse rapid and weak, and profuse cold perspiration. If the nervous depression continues, the heart fails and the patient faints, and may pass to coma and death. The danger of these symptoms may be avoided by giving some whisky or aromatic spirit of ammonia, separately or together, before the cocaine is injected. Should the patient faint, lay him down with the head lower than the chest, administer stimulants, and probably the doctor will order a hypodermic injection of strychnine, which should always be prepared in case of emergency.

General anæsthesia is produced in the general way by chloroform, ether, or nitrous oxide. A nurse should know that children or patients suffering from adenoids or enlarged tonsils take an anæsthetic very badly as a rule, as they breathe entirely through the mouth, and during or after operation profuse bleeding interferes seriously with the respiration.

Blood clots fill the pharynx or cover the epiglottis, and this can be prevented by the head hanging over the table; but if it is certain that blood has entered the larynx and impeded respiration, the surgeon will probably quickly draw the head over the end of the table, and by quick strokes on the trachea and larynx force the clots into the mouth again. The stroking or "stripping" is done from the chest to the mouth, and not vice versa. Food particles may be expelled in the same manner. For adenoid operation under cocaine the patient sits in a chair, the nurse steadying the head with her hand on the occiput. The surgeon is seated facing the patient, with the latter's knees between his.

In general anæsthesia the patient lies on the table, on his back. In adenoid operation the head should be lower than the body. If artificial light has to be used, care must be taken that any gaslight should not come near the ether cone.

Sterilization of instruments is carried on precisely as for general operations.

THE NURSES' CLINIC—*Continued.*

The simplest method is to boil them for ten minutes in a clean vessel, in water containing soda.

It is best that instruments should be used dry, and not taken out of an antiseptic solution. This point rests, of course, with the surgeon; but where no special orders have been given it is well to ask what the doctor's wishes are on the subject.

Cloths, towels, etc., used may be boiled and dried or sterilized in the usual way.

Trays, dishes, etc., should be scrubbed with hot water and soap, dried, and covered with a clean towel. The nurse should sterilize her hands once and then dip in bichloride solution when necessary, as she may be called upon to hold the lamp or touch something that is not aseptic, and would need to dip her hands in solution before again assisting at the operation. If the nurse keeps a towel wet with bichloride solution over her arm she can keep her hands sterile by having the towel round the unclean article, instead of touching it with her hands.

The most satisfactory method of sterilizing her hands is as follows: Wash the hands well with green soap and hot water, special attention being given to the nails, which should be short. The hands are then plunged into a solution of Condy's fluid, 1 in 1,000, for two minutes, afterwards rinse in solution of oxalic acid till all the brown stain is removed, and then the hands are sterile.

It is impossible to render the nose absolutely aseptic for any length of time, as it inhales microbes and is infected from the back of the throat, also from septic matter in the folds of the nasal membrane. Much, however, may be done to render it nearly sterile, if not quite so.

The nurse should first cut away the small hairs growing inside the nostril.

The nose should then be syringed out with a solution of peroxide of hydrogen and water, strength 1 to 4 or 1 to 10. After several injections, about 2 ounces at a time, a nasal douche should be given, and the nose dried with swabs and gentle blowing down by the patient. A piece of cotton wool should be put in till the surgeon begins to operate. The moustache should be carefully washed with bichloride solution 1 in 1,000. A nasal douche will generally be given hot, 108° to 112° F. To the water should be added some salt, 1 drachm to a quart of water.

After the douche is prepared it is held 6 inches above the patient's head. When the cold water has been run out, the nozzle is put into the patient's nose, on the uppermost side, the patient bending his head well on to his shoulder, over a basin, and all breathing must be done through the open mouth. The head is bent so that one side of the nose is lower than the other. The water will flow gently through one side into the back of the throat, and directly after return through the other nostril into the basin.

Then the patient blows the nose gently several times till quite clear, and then cotton wool is put in each nostril till the operation.

The mouth and pharynx then are cleansed. Cleansing the mouth is very important in operations upon the tongue, lips, and palate.

The solutions mostly in vogue are boracic acid, peroxide of hydrogen, or Condy's fluid. The teeth must be scrubbed well with a brush wet with solution, carefully cleansing between the teeth. The mouth must then be gargled frequently, and the surface of the gums and lips carefully cleaned with swabs dipped in solution. The soft palate, tonsils, uvula, and back of throat must be scrubbed gently with swabs on holders. In winding cotton wool on applicators, see that the end of the applicator is well covered by loose wool, and that the metal does not project. The free end of the wool must be loose, the other end wound firmly upon the applicator as it is turned between the fingers.

The free end will then form a pad which for the nose should be large and soft.

It is wise to cover the patient's hair during the operation with a rubber cap or towel, to prevent it infecting the surgeon's hands, and to keep the hair from getting matted with blood.

Patients should be kept on liquid food for at least twelve hours before operation if a general anæsthetic is to be given. Some beef tea may be given six hours before operation, but nothing after, not even water. The bowels should be cleared out by enema or aperient the night before.

### Incidents in a Nurse's Life.

### A BABY DRUNKARD.

A CHIRPING little yellow thing was carried into a South African hospital one winter's afternoon. It was not a sick canary bird, though in colour it resembled one. It's face, its soft, scanty hair and its shrunken body were all of a saffron hue, but the two big brown eyes were distinctly human. It was carried in the arms of a not very human-looking mother of the slum type, bare-footed, her dirty shawl and ragged skirt seeming almost to have grown on her, whilst her hair compared not unfavourably with the doormat. She appeared distinctly relieved to get rid of her burden, and bestowed it with alacrity upon the nurse. It did not "greet" when parted from "Maw," but at once transferred that appellation to anyone who wore a skirt. It mattered little if the skirt were worn by nurse, ward-maid, or dignified matron. They were all "Maw" to the little yellow thing, the new patient, whose name was Willie, and who was five years old. The brown eyes in that wizened face, with the expression of a tiny old man of three-score and ten, looked critically at everyone with the expression of a cynic, while the puckered yellow forehead seemed to consider how much drink you were good for. The criticism generally would end in "Maw! gie me a wee drap toddy!"

Willie was duly washed and placed in a crib. He never cried, but the plaintive voice from the crib was rarely quiet. The poor little restless mind wanted something, it knew not what. Willie had no respect for the presence of the chief physician. At his visit the nurses would walk about on tip-toe, the patients try to smother their coughs, and every-one be on their good behaviour. The only lawful sound was the murmuring voice of the lecturer, as he instructed his large class of students. But the well-ordered silence would be rudely and suddenly broken by a shrill treble: "Maw! bring me a pot o' beer." After a few minutes' quiet there might be a variation: "Maw! gie me a scone!" Even the great man could not be angry at the interruption. He would even smile gravely and say: "Give him anything he wants, nurse, for it will make no difference." And they all knew the verdict was passed. But the scone was just tasted and laid on the pillow, for Willie could not eat. When the nurse brought him milk and, to make him take it, told him it was the beer he longed for, the old face looked at her with scorn for attempting to deceive him. He knew beer or toddy when he saw it! His real "Maw" acknowledged he had been nourished from birth on whisky, varied with beer. "It was generally the only food going, and the 'wean' just took it with the rest."

Willie only occupied his little crib for less than a week. When the day nurses came on duty the morning of the sixth day they found it empty, for in the night Willie's little spirit had fled. He died of drink at five years old, with all the symptoms of a habitual drunkard. His enormous liver was kept as an object of instruction for the students, and his little yellow body was consigned to a pauper's grave.

### Central Midwives Board.

A MEETING of the Central Midwives Board was held on Thursday last week for the first time at Caxton House, Westminster. A distinct disadvantage of the new premises is that when the windows are open the noise of the traffic outside is so great as to render it exceedingly difficult for the speakers to be heard. The members of the Board who were present included Dr. Champneys (chairman), Miss R. Paget, Mr. Fordham, Mrs. Latter, Mr. Ward Cousins, Mr. Parker Young, and Dr. Dakin.

The minutes of the last meeting having been disposed of, a letter was read from the Clerk of the London County Council as to Dr. Wanklyn's report on the Whitechapel Union Infirmary. It was suggested that the matter be referred to the Standing Committee, but Mr. Parker Young moved that as according to Dr. Wanklyn's report the Whitechapel Infirmary was structurally unfit, and could not be recognised by the Board as a training school, no further action should be taken. This was carried, and a letter from the Local Government Board forwarding a copy of a letter from Dr. Park, the Medical Officer of Health for Dukinfield, as to the disinfection of the clothing of midwives who have been in attendance on puerperal fever, was discussed, and referred to the Standing Committee.

A question arose as to the signing of the certificate of Mrs. Carruthers, a lady who was trained at the Government Hospital, Madras. Before her term was complete the medical officer then in charge died, and the present officer, Lieut.-Colonel Sturmer, now wrote to know whether he had the authority of the Board to sign Mrs. Carruthers' certificate, not having seen her midwifery work himself. The Board decided that he had not this authority, and it appeared as if the woman would lose her certificate. It was proposed that Mrs. Carruthers should be admitted to an examination on the merits of the records of her work, which were prob ably kept at the hospital in Madras, and eventually the matter was referred to the Standing Committee.

The next matter of interest was the report of the Standing Committee. A recommendation that Dr. Walford, Medical Officer of Health for Cardiff, "be asked to state whether in his opinion the difficulty experienced by certain Welsh candidates in taking their examination in English would be met by the presence of an interpreter at the oral examination" was passed. The question of the fees of medical men called in to midwives' cases was again raised by a letter from Dr. Gordon, Hon. Secretary of the Salisbury Division. The recommendation of the committee that Dr. Gordon should be informed that the draft revision of the Rules now before the Privy Council for its approval would meet the objections raised was carried.

A question of serious import was raised in respect of two letters which stated that a certain midwife of Cardiff was in the habit of receiving pupils for training in midwifery and getting their papers—Forms III. and IV.—signed by a medical practitioner, who, it was alleged, had not personally supervised their cases. The name of a candidate who was up for the June examination was specially cited as having had her papers thus incorrectly signed. The Board agreed to the recommendation of the committee that inquiry should be made of the medical man in question.

An anonymous letter addressed to the Chairman was read, complaining of the signing of false certificates by a midwife approved for the purpose of signing Forms III. and IV., on the allegation that the pupils did not attend anything like the number of cases required by the Board for such signature By the recommendation of the committee the Board's Inspector was instructed to inspect the register of cases kept by the midwife; and a proposal was made that a register of cases attended by pupils should always be kept available for inspection.

A discussion was raised by a letter from the Registrar of the Coombe Hospital, Dublin, in reference to holding examinations in that city. The Lord President was in favour of the petition, and the suggestion was made by the Registrar of the Coombe Hospital that the cost of sending over examiners might be defrayed out of the examination fees of the candidates. This the Secretary proved to be not feasible, as it was expressly charged in the Act that the examination fee for midwives should not exceed one guinea. Mr. Ward Cousins pointed out that the Board has no power to extend the area of the operation of the Act. Finally, the draft of a letter was drawn up, signifying that the Board had carefully considered the matter. The communication proceeded to set forth that the present position by which the Board is unable to exercise any supervision over midwives practising in Ireland is unsatisfactory, expressed the opinion that an increase of fees would be contrary to the Act, and that in any case it could not cover the official expenses in London, and suggested that as it was now proposed to defray the expenses of an examination in Dublin by a special contribution, the difficulty might be met by those interested in the matter paying the travelling expenses of candidates to a centre in England.

On the application for approval as a training school of the Royal Maternity Charity of London, the Board decided that the Secretary be informed that should the Charity apply again when the work of training pupils is more thoroughly consolidated, the application would be carefully considered.

The date suggested for the next meeting was July 26.

### The Tuberculous of Paris.

### A WOMAN'S WORK TOWARDS PREVENTION.

A WELL-ATTENDED drawing-room meeting was recently held at 39 Green Street, Park Lane, at which Miss Chaptal explained the work she has done for the last six years among the very poor of a crowded district in the west of Paris. In visiting them she was much impressed by the waste of life from the ravages of tuberculosis; and she set herself to see what could be done to improve the hygiene of the people. She began in a very small way in October, 1900, by taking a shop that was used, as were one in three of the houses in the quarter, as a public-house. It was cleaned and whitewashed, a big sign was put up stating that it was a dispensary for chest diseases, and it was thrown open on three days in the week from four to seven for patients and their friends. The hours were chosen as being those most convenient for the working class. It was not desired to attract the out-of-work, nor those hopelessly ill; but those who might be cured if the disease was taken in time; and, even more, to spread the means of prevention among the people. To this end the house was made attractive, and the people made to feel they were welcome there, while Miss Chaptal and her helpers talked to them, over and over again, of the way to improve their surroundings, and to keep themselves in health. A doctor was in attendance at certain times, and food was given to the poorest; but the great feature of the scheme was the ceaseless reiteration of the first principles of health and prevention of disease. It has borne fruit, for Miss Chaptal found lately, by inquiry at the Bureau of Health, that the mortality from tuberculosis had dropped in that district from 90 per 10,000 to 49, in the years from 1900 to 1905. During those years other houses were taken, in different parts of Paris, giving equally good results.

Another part of the scheme is a steam laundry where the clothes and bed linen of consumptive patients are washed and disinfected.

Miss Chaptal has had the pleasure of starting the first training school for nurses in France.

### North-Eastern Hospital for Children.

### OPENING OF THE NURSES' HOME.

On Monday last the Lord Mayor and Lady Mayoress, together with the Sheriffs and their ladies, went in state to the North-Eastern Hospital for Children for the purpose of opening the Nurses' Home and Laundry. There was a representative assembly of guests in the marquee in the grounds of the hospital. The inhabitants of Bethnal Green and Hackney do not often have the privilege of seeing the civic coaches, with their resplendent footmen up behind, and they turned out in large numbers to enjoy the sight. Some of the most interested spectators were the small patients, who looked on from the balconies of the hospital. As soon as the Lord Mayor and his party had arrived, a tiny patient, escorted by a nurse, presented the Lady Mayoress with a lovely bouquet of pink and crimson carnations. Prayers having been read by the Rev. W. G. Morcom, Hon. Chaplain, a choir of nurses, accompanied by the band of the East London Royal Engineers, sang "All people that on earth do dwell."

Colonel Lord William Cecil, Chairman of the Committee, then cordially welcomed the Lord Mayor and Lady Mayoress, and pointed out that up to the present the nurses' quarters had been situated about a mile from the hospital. This arrangement had naturally caused much discomfort, expense, and inconvenience, and the Committee were greatly relieved to find themselves able to build the Home adjoining the hospital. The Home had cost about £8,000, all of which had been raised, but a sum of £2,500 was still needed to complete the payments for the laundry.

The Lord Mayor having declared the Home and laundry open, the Rev. G. W. Morcom pronounced the Benediction. A vote of thanks was passed to the Lord Mayor, who, in returning thanks, advised all present to do their best to collect the £2,500 still needed. The Lord Mayor then unlocked the door of the Nurses' Home, where the matron was waiting to receive the visitors and show them over the building. After this a visit was paid to the laundry, where the Lady Mayoress started the machinery for the first "wash" to be undertaken by the hospital.

The Home contains sixty separate bedrooms for the nurses—nice, airy rooms, a good height, with linoleum on the floor and a mat to step out of bed upon. The furniture and fittings are very well adapted to combine economy of space with every convenience. Besides a hanging wardrobe and hat cupboard there is a dressing-table and washing-stand combined, with drawers underneath; a writing-table, etc. The Home sisters' room, with its two windows, is a charming abode, and the two sitting-rooms—one for reading and one for music—are both very pleasant rooms, each having a large bay window overlooking the garden, with a roomy window seat, plenty of comfortable armchairs, rockers, etc. The dominant note of colour in each room is cool green. In the music-room there is a piano and a luxurious-looking sofa. There are eight bathrooms and lavatories, and, most prized of all, a shampoo-room, with spray and gas-stove for drying the hair. This is considered a unique feature, hitherto confined mostly to nurses' residential clubs. There is a lift, and the staircase on the top story leads out on to the flat roof, which will prove a charming resort on summer evenings for nurses craving a breath of fresh air. As to the laundry, it is fitted with all the latest machinery, and is arranged to wash about 10,000 articles a week. It is estimated that a saving of something like £800 a year will be effected by the erection of the laundry, and £300 by that of the Nurses' Home, being the amount of the rent hitherto paid.

### Everybody's Opinion.

### THE PRESCRIBING NURSE WE KNOW, BUT WHAT IS THIS?

The author of the article on "District Nursing in East London," which appeared in our issue of February 3, writes: In reply to "Surgeon," of Nazareth, whose letter I have just read, I imagine that skin-grafting may not be very popular in the historic city where he lives and labours, or he may have misunderstood the wording of the paragraph, and conclude that I did the grafting on my own responsibility. This was not so. For four months I persevered with the usual legitimate remedies for ulcer in vain, and because I was so desperately sorry for poor Mary Fane, I consulted a medical man who was interested in her, also matron, and they each gave me permission to do as I thought best. I am happy to say that the grafts—not the first from my arm by any means—were a complete success. I trust I may now have explained myself to the satisfaction of "Surgeon," but if he wishes I will send him the names and addresses of a few of my "grafted" cases. I also beg to state that I, too, have met the "prescribing" nurse; she is found in every community, alas!

### "ARE NURSES UNDERPAID?"

"Fair Play" writes: All district nurses owe a debt of gratitude to Mr. Pollitt for his recent speech, and as a "Queen's nurse" I should also like to thank "Common Sense" for her letter this week. Whilst heartily endorsing most of it, I would remind her that "charity begins at home." She suggests that England should assist her poorer sister Ireland. I suggest that England should first remove the beam from her own eye (in the shape of helping her own nurses), and then she will see clearly how to remove Ireland's mote. The lack of effort to make, or even help in making, any provision for her superannuated nurses is the one blot, but a bad one, in the English branch of the Queen Victoria Jubilee Institute, of which in all other respects we are so proud. A nurse belonging to it works on loyally for years, generally her best, till health fails or old age compels her retirement. Out of her modest salary she has saved what she could, and if wise has put it into the Pension Fund for investment, but this can but bring her a pittance with which to face the troubles of old age. What does she get to augment her efforts from the Institute that has had the best years of her life?—"The ever-open door!" Nothing else. She is of no more use; she can go. Verily Scotland puts us to the blush, if we are still capable of blushing, in this matter. I feel confident did our gracious patron but know and realise the state of affairs, that she would be the first to suggest and insist on a remedy.

### A PLEA FOR GOOD FEELING.

"An Irish-trained Nurse" writes: I have followed with interest, not unmixed with amusement, the correspondence recently occupying your columns *re* "Uniform," "Fever Training," and the many other topics touched upon by the various writers. One remark of your correspondent "Noblesse Oblige" so closely touches my own feeling on the subject that *esprit de corps* obliges me to thank her for the few dignified words in which, to my mind, she throws oil upon the troubled waters of the nursing world. I in no way condemn the practice of discussion on points relating to nursing interests; on the contrary, I wish there were more of it, provided it be not allowed to degenerate into petty cavilling and useless fault-finding, which, in 19 cases out of 20, leave matters in the same state as before, less a certain proportion of our professional dignity. "Noblesse Oblige" writes: "The nurses who are the heart and life of their profession . . . are those from whom I never hear a word upon any of these controversial subjects; their work is too real, too absorbing, for them to waste time and work upon superficial trivialities and the idle battle of tongues." The idea suggested by the latter portion of this quotation seems to me exactly to classify the opinions recently expressed by some of my fellow-workers. 'Tis surely but an "idle battle of tongues" for us nurses to wage war on "the use and abuse of uniform" and "the advantages and disadvantages of veils and châtelaines," whilst the very nature itself of our work is such as calls for all our energies, and should broaden our minds so effectually that no dark corners might remain in which these small petty worries could lurk. "Noblesse Oblige" is right; our work is "too real" and "too absorbing" for us to become narrow-minded. Why, because some of us have a three years' certificate, with all the advantages of modern training, should we think the less of those of our fellow-workers—for I refuse to call them anything else—who, for reasons best known to themselves, are content with a fever, mental, or maternity certificate? Then, as to the much-tortured question of uniform. There is probably no nurse who desires more earnestly than I the adoption and registration of a general uniform, with badge, to denote the training-school of, or in some cases the special branch (maternity, fever, etc.) of the profession followed by the wearer, this uniform and badge to be worn only by duly qualified persons. I strongly disapprove of the custom of dressing nursery-maids in a uniform which, although not protected by registration, is looked upon as that of a "nurse," but it seems to me that nothing can be hurriedly done to advance the one idea or to abolish the other. It is for our officers, the heads of our chief hospitals, to look into these and other matters. Important as all trifles become when considered in relation to a still more important whole, they must still rank but as "superficial trivialities" if a solid foundation be lacking to that whole. If we nurses could be sure of a perfect feeling of good-fellowship, sympathy, and loyalty among ourselves, of a respect for the profession of our choice so true that its honour becomes to us as our own, then, and only then, could we conscientiously claim a right to attempt reforms or to correct abuses, and—to my mind at least—that time has not yet come. As for those nurses who have shown themselves to be unworthy members of our profession, will not the world throw stones enough at them without our adding to the number?

### Appointments.

BATH ROYAL UNITED HOSPITAL.—Miss Constance M. Ryley has been appointed staff nurse. She was trained at the General Hospital, Great Yarmouth.

BECKETT HOSPITAL, BARNSLEY.—Miss Ethel Towers-Minors has been appointed night sister. She was trained at the District Hospital, Walsall, and the Eye Hospital, Birmingham. She has since been sister at the Royal Hospital, Sheffield, and private nurse at Great Malvern and Huddersfield.

BRADFORD CHILDREN'S HOSPITAL.—Miss Beatrice Wolfenden has been appointed staff nurse. She was trained at the General Infirmary, Macclesfield.

CANTERBURY POOR-LAW INFIRMARY.—Miss Edith Kate Bennett has been appointed superintendent nurse. She was trained at Nottingham Poor-law Infirmary, and has since held appointments at Kingston-on-Thames, Windsor, and Epsom Poor-law Infirmaries.

COTTAGE HOSPITAL, REDHILL.—Miss Rosa Copp has been appointed staff nurse. She was trained at Harrogate Infirmary, where she has since been staff nurse.

FAZAKERLEY NEW HOSPITAL, NEAR LIVERPOOL.—Miss E. M. Clarke has been appointed matron. She was trained at St. Thomas's Hospital, London, and was afterwards night superintendent and assistant matron at Gore Farm Hospital, Dartford, assistant matron at the North-Western Hospital, Hampstead, and matron of the City Hospital, Netherfield Road, Liverpool.

JESSOP HOSPITAL FOR WOMEN, SHEFFIELD.—Miss Katherine Grice has been appointed staff nurse. She was trained at the Royal Southern Hospital, Liverpool.

LAMBETH POOR-LAW INFIRMARY.—Miss Margaret Edith Okey has been appointed night superintendent. She was trained at Grimsby and District Hospital. She has since been sister at Worcester General Hospital, sister at Birmingham Poor-Law Infirmary, superintendent nurse at Cheltenham Poor-Law Infirmary, and superintendent nurse at Leek Poor-Law Infirmary. She holds the certificate of the Central Midwives Board.

LEWISHAM INFIRMARY.—Miss Robina Grigor and Miss M. I. Gordon have been appointed ward sisters. Miss Grigor was trained at Guy's Hospital, and has since been attached to the private staff. She has also been charge nurse at the Park Hospital, Hither Green. Miss Gordon was trained at the County and City Royal Infirmary, Perth. She has since been sister at the National Red Cross Hospital, South Africa, and night sister at St. Bartholomew's Hospital, Rochester. She has also done private nursing.

NORTH-EASTERN FEVER HOSPITAL, TOTTENHAM.—Miss L. B. M. Hall has been appointed charge nurse. She was trained at the Infirmary, Whipps Cross, Leytonstone, having previously received fever training at the North-Western and South-Eastern Hospital, under the Metropolitan Asylums Board; she has also done private nursing and been nurse at the Hampstead Infirmary.

NOTTINGHAM POOR-LAW INFIRMARY.—Miss Ethel Presgrave has been appointed charge nurse. She was trained at Crumpsall Workhouse Infirmary, having previously been nurse at Great Yarmouth Sanitary Authority's Isolation Hospital. She has since done private nursing.

OPEN-AIR SANATORIUM, WOKINGHAM.—Miss Gertrude Newton has been appointed staff nurse. She was trained at Essex and Colchester General Hospital, and has since been staff nurse at Dorset County Hospital and staff nurse at Seacroft Hospital, Leeds.

ROYAL EDINBURGH ASYLUM.—Miss Meta D. Blair has been appointed charge nurse. She was trained at Mavisbank Asylum, Polten, near Edinburgh, and at the Royal Edinburgh Infirmary.

ROYAL INFIRMARY, DERBY.—Miss J. V. Bishop has been appointed massage sister. She was trained at Madame Bergman Osterberg's Physical Training School and at the West End Hospital for Diseases of the Nervous System, London. She has since been masseuse at Hanover Institute for Nurses and Private Hospital, London.

ST. MARY'S HOSPITAL, PADDINGTON.—Miss M. E. Davies has been appointed matron. She was trained at King's College Hospital and the City of London Lying-in Hospital, and has since been ward sister, assistant matron, and Home sister at University College Hospital. Subsequently she has been matron of Queen Charlotte's Lying-in Hospital, London.

SAMARITAN HOSPITAL FOR WOMEN, GLASGOW.—Miss Annie Smithson has been appointed night sister. She was trained at the Chelsea Hospital for Women, London, and at the Royal Edinburgh Infirmary. She has been Queen's Nurse for three years.

TOXTETH PARK WORKHOUSE INFIRMARY, LIVERPOOL.—Miss Florence Tattersall has been appointed superintendent nurse. She was trained at the Manchester Royal Infirmary. She has since been theatre sister at Chorlton Union Infirmary and superintendent nurse at Ashton-under-Lyne. She holds the certificate of the Central Midwives Board.

VICTORIA HOSPITAL, BLACKPOOL.—Miss T. Ker has been appointed sister. She was trained at Cumberland Infirmary, Carlisle, and she has since been charge nurse at the Beckett Hospital, Barnsley; the Northern Infirmary, Inverness; and the Home Hospital, Leicester.

WALSALL DISTRICT HOSPITAL.—Miss G. Wills has been appointed matron. She was trained at the South Devon and East Cornwall Hospital, Plymouth, and has been nurse at the Newark-on-Trent Hospital and Dispensary.

### Presentations.

Toxteth Park Infirmary, Liverpool.—Miss Ellis, superintendent nurse at Toxteth Park Infirmary, who is leaving to be married to Dr. Walker, formerly house surgeon at that institution, has been presented by the staff with a case containing silver fish-carvers and a dozen fish knives and forks; also a silver tea-kettle and stand.

### The Nurses' Bookshelf.

Simple Introductory Lessons in Midwifery. By Miss M. Loane. (London: Scientific Press, 28 and 29 Southampton Street. 1s. net.)

Miss Loane explains in her Preface to this useful little book that it is intended for the use of women who ultimately desire to pass the Central Midwives' examination, but who are so entirely ignorant of the theory, and so completely unacquainted with the language in which it is usually set forth, that they cannot profit immediately by any ordinary course of lectures on the subject. The book is therefore divided into small chapters dealing with the subjects required by the Board, and having at the end of each an explanation of the technical terms used in it. Their full use would no doubt be better attained if they were read aloud to the learner, and explained by the use of a pelvis, for such words as "sacro-iliac-synchrondosis" present considerable difficulties to the class of women whom it is desirable for many reasons to attract to the profession of midwife. This could easily be done by any nurse who had passed the examination, although she might not be in the least fitted to prepare a candidate for it. The book would also be found useful for the trained nurse who intends to become a midwife, as an introduction to the larger books she must study when she is beginning in earnest. The lessons are written in as simple words as is possible on the subject; and the derivations of the terms used are given in most cases. Some guide to the pronunciation of such words as "ischium" or "œdema" would probably be found useful by the uneducated pupil, as an addition to the vocabulary.

### Lloyd's International Library.

On July 9 subscriptions to this successful undertaking of *Lloyd's News* will terminate, so that all who desire to avail themselves of a unique opportunity should lose no time in signing and sending in the order form appearing on page iii. of The Hospital.

### TRAVEL NOTES AND QUERIES.

By our Travel Correspondent.

Lodgings at Folkestone (Rose).—We do not undertake to recommend lodgings. Write to the Traffic Manager at Charing Cross, enclosing stamp, and ask him to send you their list of lodging and farmhouses. If you prefer to look for lodgings on the spot, go for a night or two to "Kentish Hotel, Alexandra Gardens," whilst pursuing your researches, or, if comfortable, you may prefer to stay there.

Neuchâtel for Three Weeks (Gertrude).—In the position of your house you would find it quite bracing, and I should recommend you to have light woollen clothing, chiefly because, though the days are hot, the evenings are often very fresh. Have one pair of stout walking boots, well nailed, for mountaineering, because, though you may do no climbing, it is a great assistance in walking on the slippery grass slopes. It is a nice neighbourhood, and there are many easy excursions.

North Wales or the East Coast (A. R. H.).—At Llanberis write to Mrs. Closs, Peris Temperance Hotel, Nant Peris; at Rhuddlan to Mrs. Ewen, Bryn Derwen, 2 Parliament Street; at Rhyl to the Misses Smith, 4 Belle Vue Terrace, Bath Street; at Yarmouth to Mrs. Edgar Bond, 42 Wellesley Road; at Felixstowe to Miss Edwards, Brierholme.

The Bay of St. Malo (Prim).—The address you want is Madame Pallot, Maison Mathias, St. Servan. There is nothing at Dinard to suit you as to terms, but you can go there daily from St. Servan in the little steamer; it is only a ten minutes' crossing, and would be quite easy for your companion.

A Fortnight on the Seine (M. T. T.).—You have not been forgotten, but earlier holidays had to be attended to first. The money at your disposal is enough, if managed with care. Go via Newhaven and Dieppe straight to Rouen. Stay there three days. Go to Hôtel du Square, 91 Rue Jeanne d'Arc. If full, go to Hôtel de la Couronne, near the Vieux Marché. After Rouen take the steamer down to Caudebec and put up at the Hôtel de la Marine. From this place you can visit all the most interesting places on the river. Return home via Havre and Southampton.

Rooms in Scotland (E. D.).—We do not reply by letter unless 2s. 6d. is sent for The Hospital Convalescent Fund. Also we do not undertake to find rooms for correspondents. Do this and you will hear of what you want: Write to the Traffic Manager at Euston and also to the Traffic Manager of the Caledonian Railway at Inverness and ask them to send you their gazettes with list of lodgings, etc. . . . I fear you will have some little difficulty in finding what you want. There is no English church, of course, at Boat-of-Garten, neither is there any Episcopalian church. There is a very nice Episcopalian service at North Ballachulist on the Caledonian Canal and a charming homely inn there kept by Mr. Carmichael. The spot is ideal, but I should not call it exactly bracing, though very healthy. I think probably living there would not be dearer than taking four rooms for yourselves, and the difficulty of the church would be overcome. You reach North Ballachulish from Oban by steamer up the canal.

Addresses at Totnes, etc. . . . (E. B.).—Many thanks for kind information sent.

A Fortnight in Belgium or Normandy (Guinevere).—Why did you not write sooner; you have left barely time for an answer? Normandy is out of the question. The sum you have at your disposal is very small, and can only be made sufficient for a holiday in Belgium. The journey there being very cheap, will just enable you to spend a fortnight there. Second-class return to Ostend from Tower Bridge will be 9s. Go first to Brussels; put up at Madame Janssen, 23 Rue de Joucker, Porte Louise. Ask for her cheapest rooms. The expense should not be more than 5 francs per day. Write to her first on a prepaid foreign postal letter card and ask her terms. Stay there five days, on one of which you can visit Mechlin and another Louraine. The excursion to Waterloo is not worth the price it costs. Then go to Ghent for two nights. One day is sufficient for the town and the next for visiting Constrai and Oudenarde. Put up at Hôtel Aux Armes de Zeelande. Ask for rooms on third floor. Then go to Bruges to Miss Le Marchand, 25 Rue de la Cour de Gand. Should her house be full (write first), go to Hôtel Panier d'Or in the Market Place. Stay at Bruges till your return. Travel always third-class; there is nothing against it, and you have only just money enough. Should it run short you must return a day earlier. No, I do not recommend personally-conducted and A1 tours in your circumstances. Cook's tickets are no cheaper; but it is a convenience to have them ready if you do not speak French.

The Seine for Three Weeks (L. E. D.).—You have not money enough to move about constantly. The only way in which you can spend three weeks on the Seine at your terms is to go to Rouen for four days, and then spend the rest of the time at Caudebec, making excursions up and down the river by steamer. Your journey out via Dieppe to Rouen will be £1 13s. 3d. second-class, return by night service, and £1 18s. 1d. by day service. At Rouen put up at Hôtel du Square, Rue Ste. Jeanne d'Arc, or Hôtel de la Couronne, near the Vieux Marché. With rooms on third floor this should not be more than 6½ frcs. Take steamer down to Candebec and go to Hôtel de la Marine. Terms 7 francs per day.

### A Book and its Story.

### A COUNTRY CHRONICLE.*

We may at once inform readers in search of the sensational that, notwithstanding the possibilities which the title of "Amelia and the Doctor" suggests in that direction, nothing in the book responds to it. No unscrupulous medico patrols its pages waxing rich from a fashionable practice among neurotic patients; nor any designing nurse laying seige to the heart of susceptible members of his profession with a view to exchanging the onerous duties of her own for those of domesticity. "Amelia and the Doctor" is merely a very every day chronicle of the doings of some of the greater people in the little village of Barton, told by a lesser member of the rustic community who looks on.

In this little circle are found the Vicar, a good parish priest, Dr. Charlton, sceptical, warmhearted, caustic-tongued; in constant, but not altogether unfriendly, antagonism with him, Miss Amelia Carey, the gentle spinster; Lord Riversdale, the landowner of the district and Colonel Fraser, V.C., retired on half-pay, each with a son and daughter. Mrs. Copman, the foster mother of Lord Riverslade's son, of whom the village stands in awe as being a woman who is indifferent to the opinion of her neighbours, plays an important part in the little drama. Then there is a burglary, a lost document, and a secret drawer in an old bureau to give a note of excitement. Never once does this rise to more than a mild flutter of expectation, for only the expected happens. There is everything in the telling of a story, and the author has not the gift of imaginative writing.

Early in the chronicle the elopement of Colonel Fraser's daughter and only child with the son and heir of Lord Riverslade lays the foundation of a romance. Lord Riverslade never saw his son again, and years afterwards, when Colonel Fraser's daughter returns a widow to die at the cottage of Mrs. Copman, she brings their child Vera with her. She had not seen her father since her elopement. She writes a last letter imploring his forgiveness, and begging that he will receive Vera for her sake. The child has been sent with the letter to her grandfather by Mrs. Copman immediately after her mother's death. His soldier servant tells him of her arrival. "At his first glance at the little girl, a child of twelve or fourteen years, the Colonel started violently. He shaded the lamp light from his eyes, the better to see the face, as the little girl came forward, apparently without fear, and handed him a sealed letter, saying, 'Please sir, mother said I was to give you this.' At the simple words the Colonel's face set itself harder and more grimly than before. 'Me, my child,' he said, 'there must be some mistake.' 'No,' the child insisted, 'mother said I was to give it to you.' He took the letter and glanced at the shaking, feeble handwriting. His leathern jaw closed, if possible, more rigidly. 'Where is your mother?' he asked. . . . The Colonel still held the letter unopened, looking at it in grim, silent thought. 'She's dead,' the child added simply. . . . 'She's dead,' she repeated. The Colonel buried his head in his two hands and groaned as he leaned forward on the table. . . . Then he lifted his head and slowly broke the seal of the envelope. The letter, after craving forgiveness, begs that he will take charge of the child Vera. 'I send you this by the hand of my child Vera, to beg you for her sake to remember that you had a daughter, and to forget, if possible, that your daughter disobeyed you. For if you will not care for my child there is no one in the wide world to care for her. . . . If you have forgiveness for me in your heart, give it all to my child.' So the letter ended. There was no signature. Then the Colonel laid the letter on the table and sat over it silently, and the great big drops fell from his eyes upon the paper. He felt a gentle touch. The child had stolen to him and laid a small hand upon his knee, looking wonderingly up into his face. . . . He laid one great bony hand over the child's small one; with the other he stroked her golden hair—and so the bond of forgiveness was sealed." He goes with the child to see her dead mother, and a search among papers left for him fails to reveal her marriage certificate. After sitting for some time apparently quite oblivious to his surroundings, he calls to Mrs. Copman and gives directions to her about Vera. "You will keep her with you to-night, Mrs. Copman; after that she will live with me." His little granddaughter puts her hand into his to say good-night, and looks up into the stern face with blue eyes widely and wonderingly open, but tender and trustful. Around Vera centres the romance of the book, which, in spite of some obstacles, ends satisfactorily.

Lord Riverslade's son being dead, the estate and title comes to his nephew, Jack Rivers. He had been educated at Eton and was a lieutenant in a cavalry regiment. Between the austere uncle and his heir strained relations exist. "Very probably his lordship did not dislike him personally, he was so handsome and gay, so well pleased with himself and the world and all it held for him. . . . But the very fact that he, and not Lord Riverslade's son, was to be the successor, perhaps, was enough to dispose his lordship against him; and, whatever the reason may have been, it is certain that Lord Riverslade delighted to wound him with caustic speeches, delivered in the most courteous, and even courtly, manner."

Lord Riverslade's daughter, "Miss Sophy," is an original and amusing character. She resents the idea that it is necessary for her to marry Mr. Jack Rivers. For one reason, she is a few years her cousin's senior, and her own inclination does not lean in his direction nor his in hers, apparently, for he tells her with effusion one day of an encounter with an unknown and "angelic" being when on a visit to Lord Riverslade. This is Vera at eighteen.

Amelia—Miss Carey—is the Lady Bountiful of Barton and the particular friend of Dr. Charlton, to whom in his most drastic moods he flies for counsel—not usually taken and generally combated. A quarter of a century before he had wished to marry her, but his pronounced political and religious views were so divergent to her own that she had not responded to his advances. One of the matters about which the gentle Amelia was in these latter days concerned was the relations existing between the Vicar, for whom she had great respect, and the outspoken doctor. The doctor shocked her orthodox mind frequently by his exchange of opinions with the Vicar. Mrs. Copman is dying, and the Vicar asks him if he considers it a duty to prolong the life of a patient suffering acutely and hopelessly beyond recovery. The answer is a characteristic one. "Considering that you orthodox people expect at your death to go to a place of infinite and everlasting joy, and think so little of this world in comparison, it is wonderful what a terrible lot of trouble you expect me to take to keep you out of it." The chronicle closes with a pretty touch of old-world romance, as Amelia and the doctor part at the door of her house on their return from Vera's wedding. "Do you think these young people are going to be happy?" he asked her. "I am sure they have every prospect of it, Richard. . . ." Then the doctor did what he had never done before—he raised the hand which he still held and kissed the fingers reverently. "In honour," he said gravely, "of the unknown Gods of the 'might have been.'"

* "Amelia and the Doctor." By H. G. Hutchinson. (Smith, Elder and Co., 6s.).

### Notes and Queries.

**REGULATIONS.**

**The Editor is always willing to answer in this column, without any fee, all reasonable questions, as soon as possible.**

**But the following rules must be carefully observed.**

**1. Every communication must be accompanied by the name and address of the writer.**

**2. The question must always bear upon nursing, directly or indirectly.**

**If an answer is required by letter a fee of half-a-crown must be enclosed with the note containing the inquiry.**

*Children's Hospital.*

(172) Can I get into a children's hospital? I am 18.—*Poppy.*

You might try the Alexandra Hospital for Hip Disease, Queen's Square, W.C., but we fear that you are too young.

*New Zealand.*

(173) Where can I get information about hospitals in New Zealand?—*B. H.*

Get "Burdett's Hospitals and Charities," or "How to Become a Nurse." The Scientific Press, 28 Southampton Street, Strand.

*A Lame Nurse.*

(174) Can a young girl who is lame become a nurse?—*H. C.*

No. A nurse should be sound in health and limb.

*Quarantine.*

(175) Is it customary for nurses to be put in quarantine when moving from scarlet fever to diphtheria wards, for instance?—*Matron.*

Certainly; thorough disinfection should be enforced when a nurse is moved from one infectious ward to another.

*A Nursing Home.*

(176) Is it necessary to register a new nursing home, and is it customary to advertise such a home?—*M. W.*

No registration of homes is yet enforced. It is certainly customary to advertise.

*Midwifery.*

(177) I am a trained nurse, and have been acting as midwife. I have failed to obtain the Central Midwives Board certificate. Can I practise?—*Newtown.*

Write for the Rules of the Central Midwives Board to the Scientific Press, 28 Southampton Street, Strand, W.C.

*Wanted a Baby to Adopt.*

(178) A district nurse knows of a respectable couple who wish to adopt a baby. Where can she advertise?—*District.*

In any of the daily papers. The Editor of "Truth," Carteret Street, Westminster, has a column for this purpose.

*Board School Education.*

(179) Will you tell me as a reader of the "Nursing Mirror," if a domestic servant with a Board School education can become a nurse?—*Anxious.*

Certainly, if she is intelligent and studious. She had better procure some nursing manuals and master their contents, and if she can do this she can apply for a vacancy at a hospital.

*Epilepsy.*

(180) Can you tell me of a home for epilepsy for a young man?—*E. A. H.*

Write to the National Society for the Employment of Epileptics, Denison House, Vauxhall Bridge Road, S.W.

*The West London Hospital.*

(181) Is the West London Hospital a training school for nurses, and how many beds has it?—*Jessy.*

Yes. It has 153 beds.

*A Screen for Dust.*

(182) I have invented a screen to fit to a window to keep out dust, and to ventilate the room. I cannot afford to patent. Do you think any of your readers might take the matter up?—*E. B.*

If your query interests our readers doubtless they will write to us, and, if so, we will forward the replies to you.

**Handbooks for Nurses.**

| | Post Free. |
|---|---|
| "A Handbook for Nurses." (Dr. J. K. Watson) ... | 5s. 4d. |
| "Nurses' Pronouncing Dictionary of Medical Terms" | 2s. 0d. |
| "Art of Massage." (Creighton Hale.) ... ... ... | 6s. 0d. |
| "Surgical Bandaging and Dressings." (Johnson Smith.) ... ... ... ... ... ... | 2s. 0d. |
| "Hints on Tropical Fevers." (Sister Pollard.) ... | 1s. 8d. |

Of all booksellers or of The Scientific Press, Limited, 28 & 29 Southampton Street, Strand, London, W.C.

### For Reading to the Sick.

LIGHT IN THE DARKNESS.

While in this lonesome world I stay,
Be Thou my Light, and Thou my Way;
No foes, no weariness I fear,
No toil, while Thou, my God, art near.

Teach me, where'er Thy Steps I see,
Dauntless, untired, to follow Thee:
O let Thy Hand support me still,
And lead me to Thy holy hill.

If rough and thorny be the way,
My strength proportion to my day;
Till toil and grief, and pain shall cease,
Where all is calm, and joy, and peace.

*John Wesley.*

Patience is the endurance of any evil, out of the love of God, as the will of God. The offices of patience are as varied as the ills of life. We have need of it with ourselves and with others; with those above and below us, and with equals; with those who love us, and those who love us not; for the greatest things and the least; against sudden trouble, and under daily burdens; disappointment as to weather or the breaking of the heart, in weariness of body, in wearing of soul; in our own failure, and others' failure to us. In all these things, from childhood's little troubles to the martyr's sufferings, patience is the grace of God, whereby we endure evil for love of Him, and keep still and motionless not to offend Him.

Patience makes the soul to be of one mind with God, and sweetens all the ills of life. It casts the light of heaven upon them, and transforms them into good. It made the bitter waters sweet, the barren and dry land fruitful. Desolation it makes loneliness with God; the parching of sickness to be the fire of His love; weakness to be His strength; wounds to be health; emptiness of all things to have all things from Him; poverty to be true riches; His deserved punishments to be His rainbow of mercy; death to be His life.—*E. B. P.*

O Thou God of Patience, give us patience in the time of trial, and steadfastness to endure to the end. O Thou Spirit of prayer, awaken our hearts, that we may lift up holy hands to God, and cry unto Him in all our distresses. O Thou gentle Wind, cool and refresh our hearts in all heat and anguish. Be our defence and shade in the time of need, our Help in trial, our Consolation when all things are against us. Come, O Thou eternal Light, Salvation, and Comfort, be our Light in darkness, our Salvation in life, our Comfort in death; and lead us in the straight way to everlasting life, that we may praise Thee, for ever. Amen.

*Bernhard Albrecht* (1569-1636).

Why restless, why so weary,
My soul, why so cast down?
Is all around so dreary,
And hath the Cross no Crown?
Where is the God Who found thee,
Who once could make thee glad?
Are not His arms around thee,
Then wherefore art thou sad?

*Clewer Manual.*

## Figures and Tables

**Fig. 16. f1:**